# Influenza vaccination during the coronavirus pandemic: intention to vaccinate among the at-risk population in the Central Catalonia Health Region (VAGCOVID)

**DOI:** 10.1186/s12875-021-01434-8

**Published:** 2021-05-01

**Authors:** Anna Bonet-Esteve, Raquel Muñoz-Miralles, Carla Gonzalez-Claramunt, Ana M Rufas, Xavier Pelegrin Cruz, Josep Vidal-Alaball

**Affiliations:** 1 Unitat de Farmàcia, Gerència Territorial Catalunya Central Institut Català de la Salut, Sant Fruitós de Bages, Spain; 2 Health Promotion in Rural Areas Research Group, Gerència Territorial de la Catalunya Central, Institut Català de la Salut, Sant Fruitós de Bages, Spain; 3 Centre d’Atenció Primària Plaça Catalunya, SAP Bages-Berguedà-Solsonès, Gerència Territorial Catalunya Central Institut Català de la Salut, CAP Plaça Catalunya. C/ Soler i March 6, 08242 Manresa, Spain; 4 Centre d’Atenció Primària Vilanova del Camí, SAP Anoia, Gerència Territorial Catalunya Central, Institut Català de la Salut, Vilanova del Camí, Spain; 5 Centre d’Atenció Primària Manlleu, SAP Osona, Gerència Territorial Catalunya Central, Institut Català de la Salut, Manlleu, Spain; 6 Universitat Autònoma de Barcelona, Barcelona, Spain; 7 Unitat de Suport a la Recerca de la Catalunya Central, Fundació Institut Universitari per a la recerca a l’Atenció Primària de Salut Jordi Gol i Gurina, Sant Fruitós de Bages, Spain; 8 Faculty of Medicine, University of Vic - Central University of Catalonia, Vic, Spain

**Keywords:** Human influenza, Influenza vaccines, Intention, COVID-19, Health Knowledge, Attitudes, Practice, Vaccination refusal, Vaccination coverage

## Abstract

**Background:**

Influenza is a major public health issue, with the primary preventive measure being an annual influenza vaccination. Nevertheless, vaccination coverage among the at-risk population is low. Our understanding of the behaviour of the influenza virus during the SARS-CoV-2 coronavirus pandemic is limited, meaning influenza vaccination is still recommended for individuals at risk for severe complications due to influenza infection. The aim of the study is to determine the intention to vaccinate against seasonal influenza among the at-risk population in the 2020-21 campaign during the SARS-CoV-2 pandemic and to analyse the factors which influence such intention.

**Methods:**

Cross-sectional telephone survey of adults (aged over 18) with risk factors in central Catalonia where the need for the Seasonal Influenza Vaccine (SIV) was recommended.

**Results:**

A total of 434 participants responded to the survey, 43.3% of whom intended to be vaccinated against influenza for the 2020-2021 influenza season, 40.8% had no intention to be vaccinated and 15.9% were uncertain or did not express their opinion. The intention to get vaccinated against influenza is associated with having dependents, the individual’s perception of the risk of being infected with influenza and the perceived risk of transmission to dependents. It is also associated with age, whether the individual had received influenza vaccine the previous season or any other season before. The best predictors of the intention to vaccinate are the individual’s perception of the risk of catching influenza and whether the individual had been vaccinated in the previous season.

**Conclusions:**

Intention to vaccinate can be a good predictor of individual behaviour in relation to vaccination. During the current SARS-CoV-2 pandemic many individuals are hesitant to influenza vaccination. In order to improve influenza vaccination coverage in people included in risk groups, it is necessary to promote educational actions, especially among those who express doubts.

**Supplementary Information:**

The online version contains supplementary material available at 10.1186/s12875-021-01434-8.

## Background

Influenza becomes a major public health problem every year as a result of the high rates of morbidity which occur during the successive waves of the epidemic and the presence of complications and excess mortality in specific population groups [1, 2].

It is estimated that influenza can affect between 5% and 20% of the general population and 50% of institutionalized populations. In previous years, it is estimated that up to 25% of acute febrile respiratory conditions could be caused by the influenza virus in Catalonia. This symptomatology generates a significant number of medical consultations and workday loss as a result of the disease and its convalescence, which can last from a few days to a few weeks [1].

In some cases, the disease can become more severe due to the influenza virus or a secondary, usually bacterial, infection which most commonly manifests itself as pneumonia, and may even prove fatal. At other times it can present in the form of cardiovascular disease, myocarditis or encephalitis, which can also be fatal. Nevertheless, frequently the initial influenza infection goes undiagnosed and is not usually recorded as the cause of death in death certificates. Overall influenza-associated mortality has been estimated to 13.8 per 100,000 person-years in Europe. Complications can occur in anyone, although hospitalizations are more common in the elderly (≥ 65 years, 309/100,000 person-years) and in young children (<1 year, 151/100,000 person-years) [3].

Influenza virus is transmissible from one day before the onset of symptoms to 4 days after. Although young children and people with weakened immune systems, might be able to infect others for a longer time. Between 30% and 50% of individuals infected with the influenza virus may be asymptomatic, though they may pass it on to others through respiratory droplets [4].

The Seasonal Influenza Vaccine (SIV) is the primary preventive measure to prevent influenza and its complications [1, 5]. In Catalonia and most neighbouring countries, it is generally recommended for at-risk populations, that is, people with underlying conditions or personal circumstances that make them more susceptible to complications or decompensation, leading hospital admissions and higher mortality rate [6].

In the Northern Hemisphere, vaccines are administered in early autumn, before the winter season begins [7]. Since the level of vaccine coverage tends to be low among the general population, due to both the strategy of specifically recommending vaccinations to the at-risk population and the low coverage achieved in this group, it is difficult for the SIV to achieve a significant decrease in influenza incidence in the community.

SIV coverage in Catalonia during the 2018-19 influenza vaccination campaign for people aged 65 years and older was 51%, similar to that of Spain (54.3%), yet in those under 60 years it was only 15.86% [1]. In people aged 60 to 64 and pregnant women the coverage was 20% and 29.2% respectively. In Spain, on the other hand, coverage was 22.3% in the 60-64 age group and 40.6% in pregnant women [8].

Influenza vaccination coverage rates fall a long way short from the 75% target proposed by the World Health Organization (WHO) and the European Centre for Disease Prevention and Control (ECDC). For the 2020-21 influenza vaccination campaign in Catalonia and Spain, the goal has been set to achieve or exceed 75% of vaccinations in people aged over 65 and in healthcare professionals, and 60% in pregnant women and people with a high risk for complications [6, 9].

According to a review of 48 articles on SIV [10], the main barriers to vaccination among the general population and even among health professionals are lack of awareness and knowledge of influenza and misconceptions about the disease and the vaccine used in its prevention.

With respect to the current SARS-CoV-2 pandemic, the 2020-21 SIV campaign is expected to be influenced by a lack of knowledge as to the behaviour of the influenza virus and the risk of coinfection by both viruses. For this reason, health authorities support an increase in SIV coverage among at-risk groups in order to reduce the number of cases which occur during the peak of the influenza season and their impact on the healthcare system [6, 9].

Behaviour, seen as a set of individual or collective actions, is a fundamental factor which influences human health. Certain behaviours related to health or illness are difficult to modify since, although they may not be unhealthy, they are thoroughly entrenched in individuals or groups. According to Ecological Systems Theory, changes in behaviour with respect to vaccination, depend on numerous factors which influence it: personal factors which make an individual accept or reject a vaccine, and also the sociocultural factors and the broader geographical and temporal context in which they live [11].

According to Ajzen's Theory of Planned Behaviour [11, 12], intention is a key determinant of action. For this reason, it is important to find out what the at-risk population’s intentions are with respect to the SIV during the current SARS-CoV-2 pandemic [13]. Also, it is relevant to examine whether the protective measures put in place to mitigate COVID-19 transmission can influence individuals in their decision to get vaccinated against influenza [14].

The aim of the study is to determine the at-risk population’s intention to vaccinate against seasonal influenza during the 2020-21 flu campaign in the Central Catalonia Health Region, in the midst of the SARS-CoV-2 pandemic. The specific objectives of the study are: 1) To assess the perception of the risk of catching seasonal influenza during the SARS-CoV-2 pandemic. 2) To determine whether contextual factors related to the increased physical and hygiene protective measures put in place during the pandemic [14] influence the intention to be vaccinated against influenza.

## Methods

This is a cross-sectional study on the intention to receive the influenza vaccine and risk perception of contracting seasonal influenza during the SARS-CoV-2 pandemic in a sample of the at-risk population by means of a telephone survey. The influence of the contextual factors related to increased hygiene/physical measures during the pandemic were also evaluated. The questionnaire used (Additional file 1) is based on an earlier survey conducted by Apiñaniz to study the acceptability of an influenza A (H1N1) vaccine [15].

The study was conducted in the Central Catalonia Health Region, which includes the counties of Anoia, Bages, Berguedà, Moianès and Osona. The duration of the study was from October 1 to 15, 2020,15 days before the start of the influenza vaccination campaign .The study population is made up of those individuals registered at the Primary Care Teams (PCTof the Catalan Institute of Health of Central Catalonia, consisting of approximately 400,000 individuals.

The inclusion criteria were: 1) Individuals aged over 18 years. 2) Eligible for influenza vaccination because of their at-risk group condition (people with underlying illnesses which make them more susceptible to complications or decompensations) (Table 1) [5]. 3) To have given written authorization to be contacted by telephone to receive health information. 4) To have given informed consent to participate in the study on a voluntary basis.

**Table 1:** Priority groups for influenza vaccination

**At-risk groups for influenza vaccination**
People aged 60 or over
People under 60 years, at high risk of complications from influenza: • Adults with chronic cardiovascular (including hypertension), neurological, or respiratory disease (including asthma, bronchopulmonary dysplasia, and cystic fibrosis). • Adults with: ○ Diabetes ○ Morbid obesity (body mass index ≥ 40 in adults) ○ Chronic kidney disease and nephrotic syndrome ○ Hemoglobinopathies and anaemia ○ Haemophilia, other coagulation disorders and chronic bleeding disorders, as well as recipients of blood products and multiple transfusions ○ Asplenia or severe splenic dysfunction ○ Chronic liver disease, including chronic alcoholism ○ Severe neuromuscular diseases ○ Immunosuppression ○ Cancer and malignant haematological diseases ○ Cochlear implant or waiting to receive one ○ Cerebrospinal fluid fistula ○ Celiac disease ○ Chronic inflammatory disease ○ Disorders and diseases that lead to cognitive dysfunction: Down syndrome, dementia and others

The exclusion criteria were: 1) A language barrier. 2) Incorrect phone number or failure to answer a call after a maximum of two attempts at different times. 3) Individuals who were uncapable of understanding the nature of the study. 4) Individuals living in institutions.

The sample size was calculated using the GRANMO calculation programme [16]. It was calculated that a random sample of 427 individuals was sufficient to estimate, with a 95% confidence level and a margin of error of +/- 5%, a population percentage which is expected to be around 51%. The replacement rate was estimated to be 10%.

The telephone survey of participants was conducted by four interviewers who had previously attended a training session for standardisation purposes and to avoid differences in the interpretation of the answers. A randomized list of individuals belonging to the study group was made available and those who met the inclusion criteria were included, until the number of participants required for the sample was obtained.

A descriptive statistical analysis was performed on the data resulting from the answers to the questionnaire. Categorical variables were described using frequencies and percentages. Continuous variables were described with mean and standard deviation. The proportions of categorical variables were compared using Fisher's exact test or Pearson's chi-square test and the t-test for continuous variables. The best fit model for logistic regression was used to predict intention to vaccinate using the Bayesian Information Criterion, only taking into account variables with significant association with intention to vaccinate For later validation of the model, the data were split into the training set (80%) and the validation set (20%).

The R Project for Statistical Computing for Windows (version 3.6.3) was used for statistical analysis. In the resulting data that were unrepresentative, levels were simulated or eliminated in order to obtain an approximate *p*-value. Results with a *p*-value higher than 0.05 (*p*<0.05) were considered statistically significant.

The study protocol was approved by the University Institute for Primary Care Research (IDIAP) Jordi Gol Health Care Ethics Committee (Code 20/177-PCV).

## Results

A total of 434 individuals with risk factors were interviewed of whom 258 (59.4%) were women. The average age of the respondents was 60 years, with an age range between 20 and 100, and 152 (35%) were under 60 years old. Four hundred and five (93.3%) participants were of Spanish origin and 206 (47.5%) were educated to primary level. Three hundred and seven participants (70.7%) lived with a partner and 217 (50.0%) were retired (Table 2).

**Table 2:** Participants’ influenza vaccination intention and sociodemographic characteristics

VARIABLE	Yes	No	DK/NO	Total	*p*
**Intention to have SIV**	188 (43.3)	177 (40.8%)	69 (15.9%)	434 (100%)	
**Gender**					0.811
Male	79 (44.9%)	71 (40.3%)	26 (14.8%)	176 (40.6%)	
Female	109 (42.2%)	106 (41.1%)	43 (16.7%)	258 (59.4%)	
Other	0 (0%)	0 (0%)	0 (0%)	0 (0%)	
**Age**					<0.001
Average	65 (12.8)	58 (12.2)	60 (13.7)		
**Age by group**					
<60	42 (27.6%)	82 (53.9%)	28 (18.4%)	152 (35.0%)	
60-70	82 (45.1%)	73 (40.1%)	27 (14.8%)	100 (23.0%)	
>70	64 (64.0%)	22 (22.0%)	14 (14.0%)	182 (41.9%)	
**Origin**					0.245
Spanish national	171 (42.2%)	168 (41.5%)	66 (16.3%)	405 (93.3%)	
Foreigner	17 (58.6%)	9 (31.0%)	3 (10.3%)	29 (6.7%)	
**Education level**					0.016*
No studies	12 (63.2%)	3 (15.8%)	4 (21.1%)	19 (4.4%)	
Primary level	98 (47.6%)	72 (35.0%)	36 (17.5%)	206 (47.5%)	
Secondary level	42 (33.6%)	63 (50.4%)	20 (16.0%)	125 (28.8%)	
Higher education	35 (42.2%)	39 (47.0%)	9 (10.8%)	83 (19.1%)	
DK/NO	1 (100%)	0 (0%)	0 (0%)	1 (0.2%)	
**Marital status**					0.697
Single	14 (36.8%)	18 (47.4%)	6 (15.8%)	38 (8.8%)	
Married/living with a partner	132 (43.0%)	124 (40.4%)	51 (16.6%)	307 (70.7%)	
Separated/divorced	15 (40.5%)	18 (48.6%)	4 (10.8%)	37 (8.5%)	
Widow(er)	27 (51.9%)	17 (32.7%)	8 (15.4%)	52 (12.0%)	
**Employment status**					<0.001*
Member of a cooperative	2 (66.7%)	1 (33.3%)	0 (0%)	3 (0.7%)	
Employer with employees	3 (30.0%)	5 (50.0%)	2 (20.0%)	10 (2.3%)	
Self-employed or employer with no employees	5 (26.3%)	12 (63.2%)	2 (10.5%)	19 (4.4%)	
Working for the family company/business	1 (20.0%)	4 (80.0%)	0 (0%)	5 (1.2%)	
Public sector employee	8 (25.0%)	14 (43.8%)	10 (31.2%)	32 (7.4%)	
Private sector employee	29 (35.5%)	52 (59.1%)	7 (7.95%)	88 (20.3%)	
Unemployed	11 (35.5%)	12 (38.7%)	8 (25.8%)	31 (7.1%)	
On furlough	1 (100%)	0 (0%)	0 (0%)	1 (0.2%)	
Retired	117 (53.9%)	67 (30,9%)	33 (15.2%)	217 (50.0%)	
Other	11 (39.3%)	10 (35.7%)	7 (25.0%)	28 (6.5%)	
**Dependents**					0.005
Children (under 14 years of age)	15 (27.8%)	29 (53.7%)	10 (18.5%)	54 (12.4%)	
Person with physical and/or mental disability > 60 years old	19 (37.3%)	17 (33.3%)	15 (29.4%)	51 (11.8%)	
None	154 (46.8%)	131 (39.8%)	44 (13.4%)	329 (75.8%)	

Fifty four (12.4%) participants had children at their care and 51 (11.8%) cared for a dependent person. Of these, 34 (32.4%) were of the opinion that this year the individuals at their care had a high risk of catching influenza, while 49 (46.7%) participants believed that they did not. One hundred and fifty-five (35.7%) respondents had been vaccinated against influenza the previous year and 216 (49.8%) had been vaccinated in another season (Table 3).

**Table 3:** Vaccination intention according to perception of risk of catching influenza, number of risk factors and vaccination history

**Perception of risk of dependents catching influenza**					0.034
Yes	15 (44.1%)	9 (26.5%)	10 (29.4%)	34 (32.38%)	
No	15 (30.6%)	27 (55.1%)	7 (14.3%)	49 (46.66%)	
DK/NO	4 (18.2%)	10 (45.5%)	8 (36.4%)	22 (20.9%)	
**Perception of the risk of personally catching influenza**					<0.001
Yes	88 (71.0%)	18 (14.5%)	18 (14.5%)	124 (28.6%)	
No	52 (25.6%)	121 (59.6%)	30 (14.8%)	203 (46.8%)	
DK/NO	48 (44.9%)	38 (35.5%)	21 (19.6%)	107 (24.7%)	
**No. of risk factors (including age)**					<0.001
	2.23 (1.26)	1.51 (0.92)	1.64 (1.03)	1.84 (1.14)	
**No. of risk factors (not including age)**	*N*=41	*N*=82	*N*=28	*N*=151	0.070
	1.34 (0.57)	1.11 (0.44)	1.25 (0.70)	1.20 (0.542)	
**Vaccinated the previous year**					< 0.001
Yes	138 (89.0%)	3 (1.94%)	14 (9.03%)	155 (35.7%)	
No	47 (17.1%)	174 (63.3%)	54 (19.6%)	275 (63.4%)	
DK/NO	3 (75.0%)	0 (0%)	1 (25.0%)	4 (0.9%)	
**Vaccinated on other occasions**					<0.001
Yes	149 (69.0%)	35 (16.2%)	32 (14.8%)	216 (49.8%)	
No	39 (17.9%)	142 (65.1%)	37 (17.0%)	218 (50.2%)	
DK/NO	0 (0%)	0 (0%)	0 (0%)	0 (0%)	

One hundred and eighty-eight (43.3%) participants reported intention to be vaccinated against influenza this season, 177 (40.8%) had no intention to be vaccinated and 69 (15.9%) did not know yet or did not answer. When asked if they intended to be vaccinated against influenza due to the context of d the SARS-CoV-2 pandemic, 212 (48.8%) answered that they did, 34 (7.8%) of whom would do so due to the pandemic and 178 (41.0%) intended to receive the vaccine regardless the context. However, 71 (16.4%) were hesitant or didn’t answer the question (Table 4). Of all those who intended to be vaccinated against influenza, 14.9% would do it due to the pandemic.

**Table 4:** Vaccination intention in light of the SARS-CoV-2 pandemic

**COVID-19 pandemic and vaccination intention**					< 0.001
Yes, regardless of the COVID-19 pandemic	154 (86.5%)	17 (9.55%)	7 (3.93%)	178 (41.0%)	
Yes, due to the COVID-19 pandemic	27 (79.4%)	1 (2.94%)	6 (17.6%)	34 (7.8%)	
No, in spite of the COVID-19 pandemic	0 (0%)	32 (97.0%)	1 (3.03%)	33 (7.6%)	
No, regardless of the COVID-19 pandemic	4 (3.39%)	111 (94.1%)	3 (2.54%)	118 (27.2%)	
DK/NO	3 (4.23%)	16 (22.5%)	52 (73.2%)	71 (16.4%)	
**Mask usage and vaccination intention**					<0.001
I will get vaccinated, even though masks offer protection,	151 (95.0%)	1 (0.63%)	7 (4.40%)	159 (36.6%)	
I will get vaccinated as masks don’t offer protection	28 (93.3%)	0 (0%)	2 (6.67%)	30 (6.9%)	
Masks don’t offer protection, but I won’t get vaccinated	0 (0%)	110 (99.1%)	1 (0.90%)	111 (25.6%)	
Masks offer protection, which is why I won’t get vaccinated	2 (4.26%)	44 (93.6%)	1 (2.13%)	47 (10.8%)	
DK/NO	7 (8.05%)	22 (25.3%)	58 (66.7%)	87 (20.0%)	
**Hand washing and vaccination intention**					<0.001
I will get vaccinated, even though hand washing offers protection.	154 (95.1%)	1 (0.62%)	7 (4.32%)	162 (37.3%)	
I will get vaccinated as hand washing doesn’t offer protection	24 (88.9%)	0 (0%)	3 (11.1%)	27 (6.2%)	
I won’t get vaccinated, even though hand washing doesn’t offer protection	0 (0%)	106 (98.1%)	2 (1.85%)	108 (24.9%)	
I won’t get vaccinated as hand washing offers protection	1 (2.0%)	49 (98.0%)	0 (0%)	50 (11.5%)	
DK/NO	9 (10.3%)	21 (24.1%)	57 (65.5%)	87 (20.0%)	
**Physical distancing and vaccination intention**					< 0.001
Social distancing offers protection, I will get vaccinated	152 (96.8%)	0 (0%)	5 (3.18%)	157 (36.2%)	
Social distancing doesn’t offer protection, which is why I will get vaccinated	24 (80.0%)	1 (3.33%)	5 (16.7%)	30 (6.9%)	
Social distancing doesn’t offer protection, nevertheless, I won’t get vaccinated	1 (0.98%)	99 (97.1%)	2 (1.96%)	102 (23.5%)	
Social distancing offers protection, which is why I will get vaccinated	1 (1.79%)	55 (98.2%)	0 (0%)	56 (12.9%)	
DK/NO	10 (11.2%)	22 (24.7%)	57 (64.0%)	89 (20.5%)	

Regarding vaccination intention and the relationship with the protective measures put in place during the pandemic, 156 (36.6%) reported they will be vaccinated despite wearing face masks and 47 (10.8%) said they won’t do so because they believe that masks already protected them. Regarding hand washing and physical distancing, 162 (37.3%) and 157 (36.2%) respondents respectively stated that they will be vaccinated despite following these measures. Fifty participants (11.5%) do not intend to be vaccinated because they believed that hand washing already protected them and 56 (12.9%) said they won’t be vaccinated due to the protection afforded by physical distancing. Aproximately 25% of respondents had no intention of getting vaccinated despite acknowledging that none of the three measures protected them. Regarding the questions related to the three contextual variables, the response Don't Know/No Opinion was chosen by 20% of the participants.

Those under 60 years of age expressed a significantly lower intention to be vaccinated than those over 60 years of age (*p* <0.001). Retired participants were more in favour of being vaccinated compared with those actively working (*p* <0.001). Living with dependents (children and adult dependents) was strongly associated with the intention to vaccinate (*p* <0.005). The intention to vaccinate was also related to the perception of the risk of catching influenza (*p* <0.001) and to the perceived risk for transmission to dependent individuals (*p* = 0.03). Those with lower levels of education were more inclined to be vaccinated than those with higher education (*p* = 0.016).

Having risk factors, excluding age, was not associated with vaccination intention (*p* = 0.07). With regard to an individual’s history of influenza vaccinations, the intention to vaccinate this year was associated with having been vaccinated the previous year and on another earlier season (*p*<0.001). Neither gender, nationality, nor marital status showed statistically significant differences with respect to the respondents’ intention to vaccinate. However, a relationship was found with their level of education and employment status.

The best logistic regression model for predicting the intention to vaccinate took into account just two variables: the subject’s perception of whether they would catch influenza and whether or not they had been vaccinated the previous year, obtaining results with a predictive accuracy of 87.7% (Table 5).

**Table 5:** Information on the logistic regression model for vaccination intention in light of the SARS-CoV-2 pandemic as a response variable

	Estimated beta coefficient	Standard deviation	Odds-Ratio	IC 95%		*P*-value
Intercept	4.709	-	-	-	-	-
Feel they will catch the influenza = NO	-1.866	0.460	0.155	0.062	0.377	<0.001
Feel they will catch the influenza = DK/NO	-1.281	0.520	0.278	0.097	0.757	0.014
Vaccinated last year = NO	-4.631	0.621	0.010	0.002	0.028	<0.001
Vaccinated last year = DK/NO	13.078	1328.368	478084.231	-	-	0.992

## Discussion

During the current SARS-CoV-2 pandemic, the 2020-21 Seasonal Influenza Vaccine campaign was perceived to be influenced by a lack of knowledge as to the behaviour of the influenza virus and uncertainty as to the degree of acceptance of SIV by the at-risk population. The health authorities have promoted increased SIV coverage, especially among the elderly and others with risk factors, to protect them and thus to also reduce the strain on the healthcare system [6, 9]. This study provides data on influenza vaccination intention and the factors which influence the intention in this population group. This makes the study of great value when planning actions aimed at improving SIV coverage in our area.

The results show that the percentage of individuals who intend to get vaccinated against influenza is similar to those who do not intend to do so, though there are also a large number of individuals who remain undecided with regard to this year’s influenza vaccination. Therefore, there is the potential for promoting health education among the vaccine hesitant or undecided, if we take into account that, according to Picazo [17], almost 50% of these who are undecided would be willing to get the vaccine if appropriately explained. Several studies show the possibility of improving vaccine coverage through educational initiatives aimed at individuals who refuse vaccines [18, 19].

According to a WHO review of barriers to influenza vaccination [11] there are no conclusive data linking age and vaccine coverage, although this study, along with some others [20], suggests that the elderly are more likely to get vaccinated. Since everyone with risk factors is encouraged to be immunized against influenza, coverage would be expected to be high [20]. Nevertheless, vaccine coverage achieved in previous campaigns [1] together with the data on vaccination intention in our study show that younger people are less vaccinated and have lower vaccination intentions.

A low perception of the risk of influenza is a major barrier to vaccination [13]. The study shows that about half of the respondents have a low perception of the risk of influenza and, in line with other studies [11, 15], this low perception is associated with the intention to refuse being vaccinated. Half of those surveyed with dependents at their care believe there is no risk for their dependents as to being infected with influenza virus and more than half of this group refuse the vaccine. This fact reveals lack of information about the disease, its chains of infections and the possible complications which make the vaccination necessary. As some studies point out, lack of awareness, misconceptions and knowledge gaps are the main barriers to influenza vaccination [10, 11].

In line with other studies, vaccination intention for the current season is high in people who were vaccinated the previous season [11, 20]. This fact shows that a history of previous influenza vaccination is a predictor of vaccination in the current season, especially if it has been a positive experience. With regard to those not vaccinated in the previous year, it is worth noting a considerable increase in the percentage of individuals intending to be vaccinated this year. There was also a significant number of hesitant patients , in keeping with the findings of the Goldman study [13].

The logistical model to predict an individual’s vaccination intention shows that, both the perception of catching influenza oneself and an individual’s vaccination history for the previous year are variables which favour influenza vaccination coverage, in line with earlier studies [11, 13, 20]. It can be seen that for levels where the response is negative or "Don't know/No Opinion", a negative relationship is maintained with the response to the vaccination intention variable. It is also noteworthy that the "Don't know/No Opinion" responses regarding vaccination in the previous year are insignificant, being almost zero. This should be taken into account in future predictions if the same model is used.

The recent pandemic has meant that users’ face-to-face access to health services and communication with health professionals has been impaired. However, organizational changes have been implemented such as the prioritization of virtual and telephone visits and the development of influenza vaccination campaigns in different spaces, outside the confines of health centres [6]. The lower degree of interaction with the healthcare system or the lack of specific and direct advice from a professional regarding vaccination appears to be related to the coverage achieved [11]. It remains to be seen how this limitation may have influenced the vaccine hesitancy and the management of indecision or doubts regarding vaccination this year.

With regard to the intention to be vaccinated against influenza this year during the SARS-CoV-2 pandemic, we found that an additional 5% coverage could be achieved with respect to the intention to vaccinate without considering this factor (question 12), a lower figure than that of the Goldman study, estimated at 10% [13]. It is known that a negative attitude towards the influenza vaccine is associated with lower vaccination rates [11] and in this study it is noteworthy that, despite the ongoing pandemic, more than a third (34.8%) of respondents reported that they would not be vaccinated regardless. These data would suggest a lack of awareness among the population regarding influenza, SARS-CoV-2 and their complications. They may also lack information regarding the risk of coinfection which, although such cases are rare, carry an increased risk for patients suffering from both infections. Hence the health authorities’ preoccupation with actively encouraging influenza vaccinations this year. Recent evidence shows that mortality in patients with SARS-CoV-2 doubles when they are also infected with the influenza virus [21].

According to a Cochrane review [22] on the effectiveness of simple physical barriers in reducing the spread of respiratory viruses, frequent hand washing can reduce the transmission of respiratory viruses; isolation and physical barriers (wearing masks, gloves and gowns) did not show a clear reduction in respiratory viral infection; and there is insufficient evidence to show that physical distancing can reduce the spread of viruses. The results show that with regard to physical protection measures (masks, hand washing and spatial separation), more than 35% of the surveyed population would be willing to be vaccinated even though they believe that the barrier already protects them. That is, they are individuals who most likely already receive the seasonal influenza shot and who are not making an exception because of the pandemic. Meanwhile, it is significant that more than 10% of the participants believe that these three measures already protect them and therefore do not intend to be vaccinated. Finally, a significant percentage of participants expressed doubts regarding the intention to be vaccinated against influenza during the pandemic and the protection offered by contextual factors. Preventive measures are effective as they are taken together rather than being mutually exclusive or implemented independently. That is why in these groups it is important to improve the information to achieve a change in attitude regarding the acceptance of vaccination.

Therefore, although the intention to vaccinate group is not much larger than the non-intention group, there are many undecided individuals, possibly due to a lack of information, misconceptions or doubts, as in other studies [10]. Although not everyone is expected to change their vaccination intention and to go on to be vaccinated, it is necessary that medical professionals take the time to uncover their doubts and reluctance regarding vaccination, in order to be able to provide them with accurate information and help them make an informed decision [23]. A recent published paper of our group showed that a brief intervention could be an effective tool in improving vaccination coverage in reluctant people [24].

This study may present certain limitations. The finding of the study may not be totally generalizable as the study has been conducted in a specific region of Spain. It did not include individuals who had not given their consent to be contacted for healthcare and research purposes; this may have led to a certain selection bias, which we attempted to minimize through the randomization of participants. Although the questionnaire employed had been adapted from a previous one, some questions, especially those related to the intention to vaccinate during the SARS-CoV-2 pandemic, had very similar answer options which could generate doubts and be difficult to comprehend. A training session was held for the interviewers in order to control for these difficulties and avoid differences in the interpretation of the answers.

To continue with this line of research, the aim is to determine the relationship between vaccination intention with regard to influenza at the start of the vaccination campaign and the number of influenza vaccinations recorded at the end of the campaign in at-risk groups.

## Conclusions

Vaccination intention can be a good predictor of people’s future behaviour with regard to the influenza vaccination, although during the SARS-CoV-2 pandemic many people are undecided, possibly due to a lack of knowledge regarding influenza, its means of transmission and possible complications. This study highlights the need to strengthen health education regarding the influenza vaccination in at- risk groups, especially in those who express vaccine hesitancy.

## Supplementary Information

**Additional file 1. **Questionnaire.

## Data Availability

Our manuscript is based on confidential and sensitive healthcare data. However, to support scientific transparency, we have posted a de-identified and appropriately redacted data for replication purposes should you or the reviewers find it helpful. The data is available at the review stage for the journal staff and reviewers at our Mendeley data repository http://dx.doi.org/10.17632/9b4b43tr9k.1

## Abbreviations

SIV: Seasonal Influenza Vaccine
WHO: World Health Organisation
ECDC: European Centre for Disease Prevention and Control
PCT: Primary Care teams
IDIAP: University Institut for Primary Care Research (Institut Universitari d’Investigació en Atenció Primària
