# Supplementary material for: Influenza vaccination during the coronavirus pandemic: intention to vaccinate among the at-risk population in the Central Catalonia Health Region (VAGCOVID)
Source: BMC Fam Pract. 2021 May 1;22:84. doi: 10.1186/s12875-021-01434-8 (PMC8087502; doi:10.1186/s12875-021-01434-8)
Supplement: Supplementary file 1 — Additional file 1. Questionnaire. [file 12875_2021_1434_MOESM1_ESM.docx]

**Appendix I. Questionnaire**

1. Good morning / good afternoon, my name is ............................ and I work at the Catalan Institute of Health . May I speak to .......................................... (name of the contact person)?

A1) If the person answering the phone is the contact Go to point B

A2) If the phone number is wrong and does not belong to the contact person. Answer: Okay, thank you for your attention. Have a nice day/ good afternoon

1. I am a contributor to a study on influenza vaccination. I contact you because you authorized us to do so over the phone. Do you want to participate in the study considering that the information you answer is completely confidential and will be recorded anonymously? Your answer will be very useful to us.

Yes Go to point C

No Answer: Okay, I still appreciate your attention. Have a nice day / good afternoon

1. Is it okay to take care of me now? I will only be with you for 5 minutes.

Yes Go to question 1

No Answer: Go to point D

1. Would you like to take care of me at another time?

Yes Go to point E

No Answer: Okay, I still appreciate your attention. Have a nice day / good afternoon

1. Can you tell me what day and time you would like me to call you? ......................................... (name of the person) Okay, so I'll do that. Thank you for your attention. Have a nice day / good afternoon

First of all I will ask you some general questions.

1) What is your gender?

Male Female Other DK/NO *Go to question 2*

2) Would you mind telling me your year of birth? *Go to question 3*

3) In what country were you born? *Go to question 4*

4) What is your highest level of education?

No studies Primary level Secondary level (GCSE or A level)

Higher education (university or vocational) DK/NO *Go to question 5*

5) What is your marital status?

Single Married or living with a partner Separated or divorced Widow(er) *Go to question 6*

6) Employment status?

Member of a cooperative Employer with employees Employee
Self-employed or employer with no employees
Working for the family company or business Public sector employee
Private sector employee Unemployed On furlough Retired Other *Go to question 7*

7) Do you have one or more of the following in your charge?

Children (under 14 years of age)

Person with physical and/or mental disability

> 60 years old

*Go to question 8 if they answer yes to one or more of the options.*

*Go to question 9 if they have no dependents*

8) Do you believe that any of the people in your charge have a high risk of catching the flu this year?

Yes No DK/NO *Go to question 9*

*9)* Do you think that you have a high risk of catching the flu this year?

Yes No DK/NO *Go to question 10*

*10) Did you get a flu shot last year?*

Yes No DK/NO *Go to question 11*

*11) Have you ever had a flu shot?*

Yes No DK/NO *Go to question 12*

*12) Do you intend to get a flu shot this year?*

Yes No DK/NO *Go to question 13*

*13) Choose the response* *which best reflects your intention to get vaccinated against the flu this year in light of the current COVID-19 pandemic*

1. I will get vaccinated, regardless of the COVID-19 pandemic.
2. I will get vaccinated due to the COVID-19 pandemic.
3. I won’t get vaccinated in spite of the COVID-19 pandemic.
4. I won’t get vaccinated regardless of the COVID-19 pandemic.
5. Don’t know/No opinion

*Go to question 15*

14) Choose the response which best reflects your intention to get vaccinated against the flu in relation to the protection against COVID-19 provided by a face mask.

1. I will get vaccinated, even though masks offer protection.
2. I will get vaccinated as masks don’t offer complete protection.
3. I won’t get vaccinated, even though masks don’t offer complete protection.
4. I won’t get vaccinated as masks offer protection.
5. Don’t know/No opinion

*Go to question 15*

15) Choose the response which best reflects your intention to get vaccinated against the flu in relation to the protection provided by hand washing.

1. I will get vaccinated, even though hand washing offers protection.
2. I will get vaccinated as hand washing doesn’t offer complete protection.
3. I won’t get vaccinated, even though hand washing doesn’t offer complete protection.
4. I won’t get vaccinated as hand washing offers protection.
5. Don’t know/No opinion

*Go to question 16*

16) Choose the response which best reflects your intention to get vaccinated against the flu in relation to social distancing.

1. I will get vaccinated, even though social distancing offers protection.
2. I will get vaccinated as social distancing doesn’t offer complete protection.
3. I won’t get vaccinated, even though social distancing doesn’t offer complete protection.
4. I won’t get vaccinated as social distancing offers protection.
5. Don’t know/No opinion

That’s it. Many thanks for taking the time to answer the questions and for participating in the study. Have a good day / good afternoon
